# Health vulnerabilities in female sex workers in Brazil, 2016

**DOI:** 10.1097/MD.0000000000030185

**Published:** 2022-09-02

**Authors:** Letícia Penna Braga, Célia Landmann Szwarcwald, Giseli Nogueira Damacena, Paulo Roberto Borges de Souza-Júnior, Inês Dourado, Ana Maria de Brito, Alexandre Grangeiro, Mark Drew Crosland Guimarães

**Affiliations:** a Postgraduate Program in Epidemiology in Public Health, National School of Public Health, Oswaldo Cruz Foundation, Rio de Janeiro, Brazil; b Health Information Laboratory, Institute of Communication and Scientific and Technological Information in Health, Oswaldo Cruz Foundation, Rio de Janeiro, Brazil; c Collective Health Institute, Federal University of Bahia, Salvador, Bahia, Brazil; d Aggeu Magalhães Institute, Oswaldo Cruz Foundation IAM/Fiocruz, Recife, Pernambuco, Brazil; e School of Medicine, University of São Paulo, São Paulo, Brazil; f Department of Preventive and Social Medicine, School of Medicine, Federal University of Minas Gerais, Belo Horizonte, Minas Gerais, Brazil.

**Keywords:** behavior surveillance, Brazil, female sex workers, health vulnerabilities, HIV infection, stigma

## Abstract

Female sex workers (FSW) suffer stigma and discrimination that negatively impact their physical and mental health and affect access to health care services. This paper aims to describe selected health indicators among FSW in 12 Brazilian cities in 2016. Brazilian cross-sectional Biological Behavioral Surveillance Survey was conducted in 2016 among 4328 FSW recruited by respondent-driven sampling. The sample weighing was inversely proportional to participant’s network sizes and the seeds were excluded from the analysis. Health indicators were estimated with 95% confidence interval and included indicators of health status, symptoms of depression, antenatal care, pap smear coverage, signs and symptoms of sexually transmitted infection, contraception and regular condom use, number of births and children alive per women, human immunodeficiency virus and syphilis testing, usual source of care, and perception of discrimination. Most participants self-rated their health as very good/good (65.8%) and 27.7% were positively screened for major depressive disorder episode on Patient Health Questionnaire-2. Antenatal coverage was 85.8% and 62.3% of FSW had access to pap smear exam in the past 3 years. A total of 67.0% of FSW were using some contraceptive method at the time of the study. Male condom was the most common method (37.1%), followed by oral pill (28.9%). A total of 22.5% FSW had never been tested for HIV and the main reasons were “not feeling at risk” (40.4%) and “being afraid or ashamed” (34.0%). The vast majority of FSW used Brazilian National Health System as their usual source of health care (90.2%). Approximately one-fifth of the participants felt discriminated against or were treated worse for being FSW (21.4%) and only 24.3% disclose their sex work status in health services. The vulnerability of FSW is expressed in all health indicators. Indicators of health status, antenatal care, pap smear coverage, and contraception were worse than in the Brazilian population, and point out to the importance of increase FSW’s access to health care services. Also, stigma and discrimination emerged as an important barrier to FSW’s health care in all dimensions and need to be struggled.

## 1. Introduction

Global priorities in the field of women’s health have experienced relevant changes during the last years. The focus formerly restricted to the health of women during pregnancy, childbirth, and postnatal period has been expanded regarding women’s comprehensive health care. That includes sexual and reproductive health, described as the state of physical, mental, and social wellbeing related to sexuality and reproduction.^[1]^

Female sex workers (FSW) are at increased risk of human immunodeficiency virus (HIV) owed to their exposure to specific higher-risk behaviors. Therefore, they have been recognized as a key population since the beginning of the acquired immune deficiency syndrome (AIDS) epidemic.^[2]^ Structural characteristics, including precarious socioeconomic conditions and low levels of education,^[3]^ as well as discrimination related to their occupation, are known to be associated with adverse health outcomes and reflected in greater vulnerability to HIV infection and other sexually transmitted infection (STI).^[4]^ Additionally, stigma and marginalization are known to negatively impact FSW’s physical and mental health and to affect the use of health care services.^[5]^

In Brazil, FSW represent 1.2% of the female population aged 15 to 49 years, corresponding to approximately half a million women.^[6]^ Several studies have assessed overall women’s health indicators,^[7,8]^ but few have focused on health indicators among FSW. Knowledge of FSW health indicators is fundamental for effective health promotion policies and for improving HIV response and STI control.

This study aims to describe health indicators among FSW in 12 Brazilian cities in 2016.

## 2. Methods

This analysis is derived from the national Biological and Behavioral Surveillance Survey (BBSS), a cross-sectional study with 4328 FSW, recruited by respondent-driven sampling (RDS) methodology.^[9]^ Due to difficulties in obtaining population-based probability samples, RDS is the most used and the preferred recruitment method to study hard-to-reach populations such as FSW.

The study was conducted in 12 Brazilian cities from July to November 2016, in order to estimate the prevalence of HIV infection, syphilis, hepatitis B and C, and to identify knowledge, attitudes toward HIV and other STIs and sexual practices among FSW. The research project was approved by the Ethics Committee of the Oswaldo Cruz Foundation (Protocol: 1.338.989). All participants signed an Informed Consent and data confidentiality was assured.

The cities were defined by the Department of Chronical Conditions and Sexually Transmitted Infections (Departamento de Condições Crônicas e Infecções Sexualmente Transmissíveis) of the Brazilian Ministry of Health according to geographical location and epidemiological relevance of HIV/AIDS in Brazil, across the 5 regions in the country: North—Manaus and Belém; Northeast—Fortaleza, Recife and Salvador; Southeast—Belo Horizonte, Rio de Janeiro and São Paulo; South—Curitiba and Porto Alegre; Central-West—Campo Grande and Brasilia The sample was preestablished by the Department of Chronical Conditions and Sexually Transmitted Infections of at least 350 FSW in each city.

Women aged 18 years or over, who reported working as a sex worker in one of the study cities, and who had at least 1 sexual intercourse in exchange for money in the past 4 months were eligible to participate in the study. Additionally, participants needed to present a valid RDS coupon, not having participated in this study edition, and could not have any signs of drug and/or alcohol consumption at the time of participation.

Before the beginning of data collection, formative research was carried out to establish the best way to implement the study in each city. Five to 10 “seeds” were chosen purposely with different characteristics (age group, race/color, socioeconomic and educational level, and work venue) to provide diversity in the sample. The seeds were FSW from the community selected during the formative research, which involved face-to-face focal groups and identified focal points and FSW leaders from nongovernment organizations. Each seed received 3 coupons to invite other FSW from their social network to participate in the study. Participants recruited by the seed received 3 coupons to recruit additional FSW from their social network, a process which was repeated until the sample reached 350 participants in each city. The fieldwork was carried out in public health units, with the exception of Rio de Janeiro and Belo Horizonte, where sites located in the city center and close to the prostitution areas were chosen for ease of access for participants.

Each FSW received a gift (small purse containing personal care products, condoms, and vaginal lubricants), payment for lunch and transportation, and reimbursement for the lost time of work as a primary incentive for their participation in the study. The secondary incentive consisted in paying R$30 for each FSW recruited by the participant and who completed their participation in the study.

The participants answered a sociobehavioral questionnaire with domains including sociodemographic characteristics and variables related to sex work activity; knowledge about HIV transmission and other STI; sexual behavior; HIV, syphilis, hepatitis B, and hepatitis C testing; use of alcohol and illicit drug use; self-rated health status; access to prevention activities and health services; discrimination and violence; and screening for symptoms of depression.

Rapid tests for HIV, syphilis, hepatitis B, and hepatitis C were performed according to the Brazilian Ministry of Health guidelines.^[10]^ Details of the implementation of the national BBSS in 2016 were described by Damacena et al.^[9]^

### 2.1. Variables

In this analysis, the following variables were used to assess the health indicators: Self-rated health status, obtained through the question: “In general, how do you self-rate your health?” categorized as “very good/good,” “regular” and “bad/very bad”; Symptoms of depression assessed by the Patient Health Questionnaire-2 (PHQ-2) scale: the proportions of FSW with little interest or pleasure in doing things and those feeling down, depressed or hopeless were calculated. The PHQ-2 score ranges from 0 to 6 and cutoff point of 3 was considered as indicator of a positive screening for major depressive disorder^[11]^; Antenatal care indicators, related to the last pregnancy: antenatal coverage, proportion of women who initiated antenatal care in the first trimester of pregnancy, proportion of women who had 7 or more antenatal consultations, and adequate antenatal care, calculated from the combination of the last 2 indicators, that is, the proportion of women with 7 or more antenatal consultations, beginning in the first trimester of pregnancy. The last 3 indicators were among women who gave birth within 2 years prior to the research; Pap smear coverage calculated through the frequency of FSW who had pap smear exam in the last 3 years. Signs or symptoms of STI considered the occurrence of at least 1 sign or symptom: lesions, blisters or warts in the anus or vagina on the last 12 months; Signs and symptoms of STI: the occurrence of at least 1 sign or symptom, lesions, blisters or warts in the anus or vagina, on the last 12 months; Contraception: number of births and children alive per women, proportion of women who made tube sterilization (among those who do not use any contraceptive method); proportion of women using contraception and type of contraceptive method used. Also, the proportion of FSW with up to 2 childbirths and the proportion of FSW with up to 2 children alive (among FSW aged 18–49 years) were calculated and compared with the Brazilian female population; Consistent condom use with steady partners and clients, defined as condom use every time during vaginal sex in the past 6 months; HIV and syphilis testing: proportion of FSW who had never been tested for HIV; proportion of FSW who tested in the 12 months before the survey; proportion of FSW who tested >1 year before the survey; proportion of FSW who performed the last test for HIV in public services (among those who have been tested for HIV at least once in their lives); and proportion of FSW who have never been tested for syphilis. Among the FSW who never tested for HIV, the main reasons for not taking the test were also analyzed; Usual source of care: obtained through the question “Where do you usually go when you need health care assistance?,” classified as public health services from the Brazilian National Health System (Sistema Único de Saúde) and private health care; Perception of discrimination in health services was estimated based on the question: “Have you ever felt discriminated against or treated worse than other people in the health services for being a female sex worker?”; and, Disclosure of FSW status to health care providers—yes and no.

### 2.2. Data analysis

Statistical analysis considered the complex sampling design of RDS recruitment, taking into account the dependence of the observations, resulting from the recruitment chains, and the unequal selection probabilities, due to different network sizes of the participants.^[12]^ The social network size of each participant was obtained through the question: “How many sex workers who work in this city do you know personally, that is, that you know them and they know you?” The sample weighing was inversely proportional to the network size of each participant^[9]^ and the seeds were excluded from the analysis, as proposed by Salganik and Heckathorn.^[13]^

Point estimates and 95% confidence intervals were calculated for all health indicators described above. Qui-square test was performed to compare sociodemographic variables and access to antenatal care and Pap smear exams. All statistical analyses were performed using the SPSS software version 21.0 for complex samples.

## 3. Results

Among the 4328 FSW recruited, 83 seeds were excluded from the analysis. From the total sample of 4245 FSW, 49.7% were under 30 years (mean: 32.0, standard deviation: 0.3), 46.6% worked at street points, 47.8% had incomplete middle school, and 38.7% started sex work before the age of 18 (mean: 20.8, standard deviation: 0.2; Table 1).

**Table 1:** Descriptive analysis of sociodemographic and sex work characteristics among female sex workers in Brazil, 2016.

Variables	n	Percentage (%)	95% CI
Sociodemographic			
Age (yr)			
18–29	2110	49.7	47.6–51.9
30–39	1118	26.3	24.6–28.1
40–49	650	15.3	13.9–16.8
≥50	366	8.6	7.6–9.8
Educational level			
Never studied/incomplete elementary school	626	14.9	13.5–16.4
Complete elementary/incomplete middle school	1386	32.9	31.1–34.8
Complete middle school/incomplete high school	1095	26.0	24.3–27.8
Complete high school or more	1103	26.2	24.5–28.0
Sex work			
Work place			
Street points	1977	46.6	44.5–48.7
Others	2264	53.4	51.3–55.5
Age began sex work (yr)			
≤ 13	335	8.0	6.9–9.1
14–17	1293	30.7	28.9–32.6
≥18	2580	61.3	59.3–63.3

CI = confidence interval.

Most participants self-rated their health as very good/good and less than one-third, regular. More than 10% of FSW had little interest in doing things or were feeling down nearly every day in the past 2 weeks while almost one-third of FSW screened positive (PHQ-2 ≥ 3 points) for potential major depressive disorder (Table 2).

**Table 2:** Descriptive analysis of self-rated health and symptoms of depression among female sex workers in Brazil, 2016.

Variables	n	Percentage (%)	95% CI
Self-rated health			
Very good/good	2770	65.8	63.9–67.6
Regular	1259	29.9	28.2–31.7
Bad/very bad	182	4.3	3.6–5.2
Symptoms of depression – past 2 wk			
Little interest or pleasure in doing things			
Not at all	1975	47.5	45.6–49.5
Several days	1268	30.5	28.7–32.4
More than half the days	369	8.9	7.8–10.1
Nearly every day	543	13.1	11.8–14.4
Feeling down, depressed or hopeless			
Not at all	1854	44.5	42.6–46.5
Several days	1339	32.2	30.4–34.1
More than half the days	372	8.9	7.8–10.1
Nearly every day	597	14.3	13.0–15.8
PHQ-2			
Positive: major depressive disorder is likely	1144	27.7	29.5

CI = confidence interval, PHQ-2 = Patient Health Questionnaire-2.

Antenatal coverage was 85.8%, and most of FSW initiated antenatal care in the first trimester of their pregnancy. A proportion of 59.3% had 7 or more antenatal consultations, and half of them had adequate antenatal care, in terms of number of consultations and beginning of antenatal care.

A total of 62.3% of FSW had access to pap smear exam in the past 3 years. Less than one-third of FSW had signs or symptoms of STI in the past 12 months and the most frequent sign was lesion in anus or vagina. Regarding contraception, 14.0% of FSW were sterilized through tube sterilization, and 67.0% were currently using some contraceptive method. Male condom was the most common method, followed by oral pill. The least frequent methods were intrauterine device (IUD) and female condom. About one-third of FSW reported consistent condom use in vaginal sex with steady partners in the past 6 months. This percentage was 80.5% for clients.

Concerning the number of childbirths, 66.5% of FSW had up to 2 childbirths and 59.9% had up to 2 children alive. Women aged 30 to 39 years had the highest percentage of >2 births and >2 children alive (Table 3).

**Table 3:** Descriptive analysis of antenatal care, pap smear, signs and symptoms of sexually transmitted infections, contraception, condom use, and number of births among female sex workers in Brazil, 2016.

Variables	n	Percentage (%)	95% CI
Antenatal care			
Antenatal coverage	561	85.8	82.1–88.8
Antenatal care in the 1st trimester	424	63.9	59.1–68.5
≥7 antenatal consultations	327	59.3	53.6–64.8
Adequate antenatal care	292	52.9	47.2–58.5
Pap smear exam in the past 3 yr	2607	62.3	60.4–64.3
Signs and symptoms of STI			
Any signs or symptoms	432	10.3	9.1–11.6
Lesions in anus or vagina	257	6.1	5.2–7.1
Blisters in anus or vagina	199	4.7	3.9–5.7
Warts in anus or vagina	129	3.1	2.5–3.8
Contraception			
Tube sterilization	595	14.0	12.7–15.4
Use of contraceptive method	2807	67.0	65.0–68.9
Type of contraceptive used			
Oral pill	806	28.9	26.8–31.2
Male condom	1033	37.1	34.8–39.5
Female condom	32	1.2	0.7–1.9
Injectable contraceptive	783	28.1	25.9–30.4
IUD	44	1.6	1.1–2.2
Other	87	3.1	2.4–4.1
Regular condom use			
Steady partners	854	34.8	32.3–37.4
Clients	3356	80.5	78.8–82.1
Number of births per women			
≤2	2677	66.5	64.6–68.4
Number of children alive			
≤2	1708	59.9	57.5–62.1

CI = confidence interval, IUD = intrauterine device, STI = sexually transmitted infections.

Results related to HIV and syphilis testing, source of health care, and perception of discrimination among FSW are shown in Table 4. A total of 38.9% of participants tested for HIV in the last 12 months before the survey, while 22.5% had never been tested for HIV. The main reasons for never having been tested for HIV were “not feeling at risk” and “being afraid or ashamed.” Most women reported having undertaken their last HIV test in the public health service. Almost half of the women had never had a syphilis test.

**Table 4:** Descriptive analysis of HIV and syphilis testing, source of care, and perception of discrimination among female sex workers in Brazil, 2016.

Variables	n	Percentage (%)	95% CI
HIV and syphilis testing			
Had HIV testing in			
<1 yr	1642	38.9	37.0–40.9
≥1 yr ago	1592	37.8	35.9–39.7
Never	947	22.5	20.9–24.2
Reasons for never tested for HIV			
Do not feel at risk	375	40.4	36.2–44.7
Being afraid or ashamed	315	34.0	30.0–38.2
Do not know where to take the test	79	8,5	6,3–11,4
Place of last HIV test			
Public services	2411	74.7	72.8–76.6
Never had syphilis testing	2019	47.6	45.6–49.6
Usual source of care			
SUS	2689	90.2	88.7–91.5
Private care	292	9.8	8.5–11.3
Perception of discrimination			
Perception of discrimination in health services for being FSW	893	21.3	19.8–23.0
Disclosure FSW status in health services	1014	24.3	22.7–25.9

CI = confidence interval, HIV = human immunodeficiency virus, SUS = Brazilian national health system, FSW = female sex worker.

The vast majority of FSW used Brazilian National Health System (SUS) as their usual source of health care (90.2%). Approximately one-fifth of the participants felt discriminated against or were treated worse for being FSW and only a quarter disclosed sex work status in health services.

Regarding qui-square test results (Table 5), FSW with younger age, higher education, and higher income had greater access to antenatal care. The result was statistically significant for educational level and income, which almost 90.0% of FSW with complete elementary school or more and 93.9% with income >US$410 accessed antenatal care. For Pap smear exam, the result was statistically significant for all sociodemographic variables. FSW with >35 years, complete elementary school or more and receiving >US$410 per month had a Pap smear exam in the past 3 years.

**Table 5:** Qui-square test for sociodemographic variables versus antenatal care and Pap smear exam among female sex workers in 12 Brazilian cities, 2016.

Sociodemographic variables	Antenatal care			Pap smear		
	n (%)		*P* value	n (%)		*P* value
	yes	no		yes	no	
Age (yr)						
18–35	475 (86.8)	84 (13.2)		1550 (60.2)	984 (39.8)	
>35	60 (75.4)	12 (24.6)	.091	1096 (66.2)	547 (33.8)	.003
Educational level						
Never studied/incomplete elementary school	53 (74.3)	23 (25.7)		333 (52.9)	258 (47.1)	
Complete elementary or more	480 (87.7)	72 (12.3)	.013	2304 (64.1)	1268 (35.9)	.001
Income						
≤$410	434 (84.8)	82 (15.2)		1997 (60.5)	1239 (39.5)	
>$410	82 (93.9)	9 (6.1)	.039	549 (70.9)	239 (29.1)	<.001

## 4. Discussion

The data reported in this analysis indicates that FSW are at a high vulnerability, stigma, and discrimination potentially associated with their occupation.

The proportion of FSW who self-rated their health as very good or good was lower (65.8%) than the percentage found in the Brazilian female population with the same age composition (71.5%), estimated with data from the National Health Survey (Pesquisa Nacional de Saúde [PNS]), 2013.^[14]^ One-third of FSW screened positive for PHQ-2, similar to the proportion found in Southern India, of 29.0%.^[15]^ We should note that PHQ-2 is a scale for depressive symptoms screening, not for diagnosis of depression. Also, considering the increasing prevalence of major depressive disorder in the Brazilian population (5.0%),^[16]^ further evaluation among FSW is needed using more accurate diagnosis tools of major depressive disorder.

Incomplete education and material difficulties have been shown to be associated to deterioration of health perception in female population in Brazil.^[17]^ For mental health problems in FSW, a systematic review in low- and middle-income countries pointed out poverty, low education, violence experiences, alcohol and illicit drug use, inconsistent condom use, and HIV infection as key risk factors..^[18]^ In the national BBSS, the high vulnerability profile found points out to worse self-rated health and to high prevalence depressive disorders in FSW.

Antenatal coverage among FSW, of 85.8%, was far below the antenatal coverage in the Brazilian female population (97.9%), and lower than that estimated among illiterate women (89.4%), according to data from National Information System on Live Births in Brazil, in 2016 (Sistema de Informações Sobre Nascidos Vivos).^[19]^ Additionally, it was a little lower than the antenatal coverage among Brazilian indigenous women (86.6%), estimated with data from the National Survey on Health and Nutrition of Indigenous People.^[20]^ In addition, antenatal coverage in Brazilian FSW was lower than the coverage estimated in a study in South Africa, of 90.0%,^[21]^ and lower than the 1 in Bangladesh, of 91.8%.^[22]^ The indicator of adequate antenatal care (beginning in the first trimester and 7 or more antenatal consultations) also showed lower proportions than in the Brazilian female population.^[8]^ Antenatal care is important to reduce the risk of maternal and child death during pregnancy and childbirth and it provides opportunities for regular checkups, which include HIV and other STI testing. Hence, antenatal is an entry for health services for HIV testing and linkage to HIV care.^[1]^

Pap smear coverage in the past 3 years in FSW (62.3%) was far below the Brazilian target of 85% by 2022.^[23]^ It was also less than the proportion found in the Brazilian female population of 79.4% (95% confidence interval: 78.5–80.2).^[7]^ However, pap smear coverage among FSW in Brazil was greater than in China and South Africa, where the frequency of FSW who ever had a pap smear were 15.3% and 29.0% respectively.^[24,25]^ Periodic gynecological exam is important to STI diagnostic and cervical cancer prevention and should be part of comprehensive health care among FSW.

The most common symptom of genital herpes were lesions in vagina or anus.^[26]^ The high frequency of this symptom in our study may indicate that the prevalence of genital herpes could be high among the FSW studied. The overall prevalence of STI signs and symptoms (10.3%) in FSW was lower than that found among Brazilian men who have sex with men (MSM) (34.1%)^[27]^ and among HIV-negative MSM from Germany (30.15%).^[28]^ It can be partially explained by the fact that in our study, signs and symptoms were self-reported, compared to indicators in these 2 studies, which included serological data among asymptomatic MSM.

Additionally, worldwide, prevalence of human papillomavirus (HPV) infection is high^[29]^ and high burden of HPV is expected in FSW.^[30]^ Considering the proportion of 10.3% of FSW who presented signs or symptoms of STI in the past year, including those related to HPV infection, treating warts and cervical intraepithelial neoplasm associated with HPV is necessary to prevent the progression to cervical cancer.^[31]^ Also, it should be emphasized the importance of HPV vaccination among Brazilian adolescents.^[32]^ HPV vaccination was incorporated into the Brazilian immunization program in 2014 for girls aged 9 to 14 years and for women living with HIV/AIDS, aged 9 to 26 years.^[33]^ With wide vaccination coverage, a better scenario is expected, with lower HPV burden in FSW.

The proportion of FSW using any contraceptive method is less than that found in the Brazilian population by 13 percentage points. The type of method used differs from the Brazilian female population profile: while oral contraceptive is the most used method by the Brazilian female population, male condom is the most used by FSW.^[34]^ The proportion of FSW who use condoms in all vaginal sexual intercourse with steady partners was low, of 34.8%, and with clients, much higher, of 80.5%, indicating the influence of condom use by FSW for prevention HIV infection and other STI and the poor access to contraceptive methods. Although IUD is an effective contraceptive method available free of charge in primary health care in Brazil,^[35]^ the frequency of IUD use was low, approximately 6 times lower than in Russia, where 9.0% of FSW choose this contraceptive method.^[36]^ This result followed the Brazilian profile, in which IUD is used by <2% of the Brazilian women of reproductive age.^[34]^

The proportion of FSW who had up to 2 childbirths (66.5%) was similar to the proportion of childbirths among Brazilian women aged 18 to 49 years, estimated in PNS-2013 (69.9%) (PNS, 2013). However, the proportion of FSW with up to 2 children alive (59.9%) was lower than the one estimated in PNS-2013 (73.2%). This contrast can be explained by the difference in the age composition between the surveys: while most FSW were 18 to 24 years (36.9%), most women in PNS-2013 were 35 to 44 years (38.1%). Also, it is possible to assume that the number of child who have died is greater among FSW.

The proportion of FSW who ever tested for HIV in the total sample (77.5%) was higher than the percentage found in the national BBSS in 2016 among MSM (66.2%).^[37]^ The higher coverage of HIV testing during the lifetime among FSW is probably explained by the performance of HIV testing in antenatal care. Unlike men, women associate HIV testing as one of the elements of health care assistance related to pregnancy and not to their risk exposure.

An important reason for not taking the HIV test was “being afraid or ashamed,” indicating the stigma and discrimination as barriers to HIV diagnosis.^[38]^ Furthermore, in the total sample, 1 in 5 FSW felt discriminated against or treated worse for being FSW in health services. The feeling of discrimination influences women to not disclose their sex work status at health services, influencing HIV response.^[4]^

It is known that having a regular source of care is an important predictor of health care utilization.^[5]^ A substantial proportion of FSW use SUS as usual source of care, in which the family health strategy or basic health units are the most used services (53.5%). Whereas the discrimination associated with FSW is a critical barrier to access to health,^[4]^ those services should drive actions to avoid loss of opportunities for prevention, linkage, and retention to care.

Qui-square test results pointed out that structural determinants and the overlapping vulnerabilities play an important role in access to health care by FSW. It raises hypothesis that should be better explored in other analysis, considering different levels of determinants. Also, in-depth analyses of the barriers to access health care services by FSW could support target interventions.

The limitations of this study refer to the RDS method for FSW recruitment. One of the factors that influence the development of the networks is the homophily effect, defined by the participant’s tendency to recruit their peers with similar characteristics to hers/his.^[39]^ The payment of incentives also influences the network’s development, encouraging the participation of people with lower income and more socially marginalized.^[40]^ The place selected to carry out the study may also influence the network composition, according to the characteristics of the FSW who work in the proximities.^[9]^ In some cases, this resulted in overrepresentation of crack user FSW, for example, in São Paulo and Salvador; in others, in underrepresentation of street point FSW, such as in Belo Horizonte.^[41]^ Another limitation refers to the delay between the study time frame and the publication. Despite this delay, the results represent relevant data regarding health vulnerability among FSW and are still valid in view of the scarcity of large studies among this population in Brazil.

The results reported in this study are relevant for planning health policies for FSW. The social vulnerability is expressed in all health indicators considered in the study. Furthermore, stigma and discrimination emerged as an important barrier to effective women’s health care in all dimensions. Considering the vast majority of FSW in our study use SUS as usual source of care, the findings highlight the importance of guaranteeing comprehensive women’s health in the public health system, linking mental health services to HIV, STI, and sexual and reproductive health programs. Health professionals should be sensitized, and services should to be adapted to address FSW needs and to not lose opportunities for prevention and linkage to care. FSW’ community should be mobilized to fight for their rights and target actions to end stigma and discrimination.

## Acknowledgments

The authors thank the participants of the study and to the local teams that carried out the fieldwork in the 12 cities. The authors also thank the support of Department of Chronical Conditions and Sexually Transmitted Infections of the Brazilian Minister of Health; additionally, authors thank the support of The Brazilian FSW Group: Célia Landmann Szwarcwald, Paulo Roberto Borges de Souza Júnior, Orlando C. Ferreira Jr., Giseli Nogueira Damacena, Neide Gravato da Silva, Rita Bacuri, Helena Brigido, Hermelinda Maia Macena, Ana Brito,Inês Dourado, Mark Drew Crosland Guimarães, Wanessa da Silva de Almeida, Alexandre Grangeiro, Carla Luppi, Karin Regina Luhm, Isete Maria Stella, Adriana Varela Espinola,Tânia Varela, Francisca Sueli da Silva.

## Author contributions

All authors took part in the concep and the study design, data analysis and interpretation, drafting the intelectual contents and approving the final version of the manuscript.
